# Implementierung von künstlicher Intelligenz (KI) im Gesundheitswesen: Historische Entwicklung, aktuelle Technologien und Herausforderungen

**DOI:** 10.1007/s00103-025-04086-6

**Published:** 2025-06-25

**Authors:** Jill von Conta, Merlin Engelke, Fin H. Bahnsen, Amin Dada, Elisabeth Liebert, Felix Nensa, Jens Kleesiek, Anke Diehl

**Affiliations:** 1 Institut für Künstliche Intelligenz (KI) in der Medizin (IKIM), Universitätsmedizin Essen, Essen, Deutschland; 2 Institut für Diagnostik und Interventionelle Radiologie und Neuroradiologie, Universitätsmedizin Essen, Essen, Deutschland; 3 Department für Digitale Transformation, Universitätsmedizin Essen, Universität Duisburg-Essen, Essen, Deutschland

**Keywords:** Künstliche Intelligenz (KI), Gesundheitswesen, Medizin, Digitalisierung, Technologie, Artificial intelligence (AI), Healthcare, Medicine, Digitalization, Technology

## Abstract

Die historische Entwicklung der künstlichen Intelligenz (KI) im Gesundheitswesen seit den 1960er-Jahren zeigt eine Transformation, die von einfachen regelbasierten Systemen zu komplexen, datengetriebenen Ansätzen reicht. Frühe Anwendungen konzentrierten sich auf Entscheidungsunterstützung, während innovative Systeme neuronale Netze und maschinelles Lernen nutzen, um Muster in großen Datensätzen zu erkennen. Die Integration von KI-Technologien in der Medizin hat vielfältige Anwendungsfelder hervorgebracht, die sich in präventive, diagnostische, KI-gestützte Therapie und administrative KI unterteilen lassen. Präventive KI analysiert Risikofaktoren, um frühzeitige Interventionen zu ermöglichen, während diagnostische KI zu schnelleren und präziseren Diagnosen beiträgt. KI-gestützte Therapie unterstützt individualisierte Behandlungen, etwa durch personalisierte Medikation. Administrative KI optimiert Prozesse wie Terminplanung, Ressourcenmanagement und Abrechnung.

Trotz ihrer Potenziale stehen KI-Systeme vor Herausforderungen. Dazu zählen die Fragmentierung von Gesundheitsdaten, mangelnde Standardisierung, Datenschutzbedenken und algorithmische Verzerrungen. Der Aufbau interoperabler Dateninfrastrukturen und die Entwicklung ethischer Leitlinien sind entscheidend, um diese Hürden zu überwinden. Zukünftige Trends umfassen die Weiterentwicklung von Foundation Models (großen KI-Modellen, die auf breiten Datensätzen basieren und vielseitig einsetzbar sind), die Integration strukturierter und unstrukturierter Daten sowie eine stärkere Personalisierung in der Medizin. Langfristig kann KI die Qualität und Effizienz der Gesundheitsversorgung verbessern. Voraussetzung dafür sind jedoch enge Kooperationen zwischen Anwendern, Forschung, Industrie und Politik, um eine sichere und nachhaltige Implementierung zu gewährleisten.

## Abstract (en)

The historical development of artificial intelligence (AI) in healthcare since the 1960s shows a transformation from simple rule-based systems to complex, data-driven approaches. Early applications focused on decision support, while innovative systems use neural networks and machine learning to recognise patterns in large datasets. The integration of AI technologies in medicine has given rise to diverse areas of application, which can be categorized into preventive AI, diagnostic AI, AI-assisted therapeutic, and administrative AI. Preventive AI analyses risk factors to enable early interventions, while diagnostic AI contributes to faster and more accurate diagnoses. AI-assisted therapy supports individualized treatments, such as personalized medication. Administrative AI optimizes processes such as appointment scheduling, resource management and billing.

Despite their potential, AI systems face challenges. These include the fragmentation of health data, a lack of standardisation, data protection concerns and algorithmic biases. The establishment of interoperable data infrastructures and the development of ethical guidelines are crucial to overcoming these hurdles. Future trends include the further development of foundation models (large AI models that are based on broad datasets and can be used in a variety of ways), the integration of structured and unstructured data and greater personalisation in medicine. In the long term, AI can improve the quality and efficiency of healthcare. However, this requires close co-operation between research, industry and politics in order to ensure safe and sustainable implementation.

## Einleitung

Die Forderung nach Anwendungsentwicklungen und einem zunehmenden Einsatz von künstlicher Intelligenz (KI) in der Medizin ist allgegenwärtig. Ziel ist es, Gesundheitsdaten effizient, präzise und zum Erhalt einer qualitativ hochwertigen Gesundheitsversorgung auszuwerten. Vor dem Hintergrund der demografischen Entwicklung mit einer Zunahme an Krankheitslast in der Bevölkerung bei sich gleichzeitig verschärfendem Fachkräftemangel könnte KI zur Verbesserung von Prävention, Diagnostik und Therapie genutzt werden. Die sehr schnelle KI-Entwicklung in anderen Sektoren und ihre wachsende Anwendung im Gesundheitswesen werfen zentrale Fragen zu Herausforderungen und Lösungsansätzen auf. Trotz ihres enormen Potenzials stellt sich daher die Frage: Warum wurde KI im Gesundheitswesen bislang nicht umfassend genutzt?

Spätestens seit der Entwicklung leistungsfähiger KI-Grundlagenmodelle – insbesondere großer Sprachmodelle (Large Language Models) und sogenannter Foundation Models, die als breit einsetzbare Basismodelle für verschiedenste Aufgaben dienen – kann der kontinuierlich wachsende Einfluss von KI auf nahezu alle Arbeitsbereiche, inklusive Alltagsprozesse, beobachtet werden. Bereits 2019 entwickelte OpenAI das Sprachmodell GPT‑2, das den Grundstein für die Entwicklung von Content, Übersetzungsdiensten und Chatbots in vormals unvorstellbarer Qualität legte. Woran liegt die schleppende Adaption im Gesundheitsbereich? Für technische Entwicklungen sind große Mengen an Trainingsdaten erforderlich, deren Generierung in der Industrie oder für Anwendungen wie die Bildanalyse häufig unproblematisch ist. Gesundheitsdaten jedoch stehen nicht nur unter einem strengen Datenschutz, sondern sie werden in unterschiedlichen klinischen Systemen erzeugt und gespeichert, wobei überwiegend Standardisierung, Strukturierung und Interoperabilitätsprobleme eine spätere Datenaggregation verhindern. Eine erfolgreiche Entwicklung von KI in der Medizin bezog sich daher bisher zumeist auf die monozentrische Nutzung der Daten einzelner Kliniken oder Krankenhäuser, wie zum Beispiel die Auswertung von radiologischen Bilddaten. In der Radiologie wurde schon vor über 30 Jahren der DICOM-Standard international eingeführt, wodurch standardisierte Trainingsdaten zur Verfügung standen, die die Entwicklung zahlreicher KI-gestützter Bildauswertungstools ermöglichten.

Während die ersten KI-Systeme noch mit wenigen Tausend Datensätzen entwickelt wurden, werden für die Entwicklung von generativer KI einige Terabytes genutzt. Die fortschreitende Digitalisierung führt zu einem Anstieg von Gesundheitsdaten, der die Weiterentwicklung von KI-Applikationen vorantreibt, deren Einsatz an interoperable Datennutzung gebunden ist. Datenstruktur und -verfügbarkeit sind entscheidend für die Entwicklung von KI-Anwendungen in der Medizin. Historisch verzögerte sich diese Entwicklung aufgrund des gesetzlichen Schutzes von Gesundheitsdaten und der langsamen Digitalisierung im medizinischen Bereich. Zudem erschwert das Fehlen einer gemeinsamen Dateninfrastruktur, -standardisierung und -aggregation den Fortschritt. Datengetriebene, lernende Systeme könnten jedoch komplexe Krankheitsprognosen und Therapieunterstützung leisten, wenn sie hierfür Informationen aus unterschiedlichen Datenquellen kombinieren könnten, wie beispielsweise Informationen zu Krankheitsverlauf, Bilddatenanalyse, medikamentöse Therapie, klinische Symptomatik und neueste wissenschaftliche Erkenntnisse. Wenn die Vorbedingung einer interoperablen Nutzung von Daten aus verschiedenen klinischen Systemen und ggf. Gesundheitssektoren erfüllt würde, könnte ein bahnbrechender Fortschritt in Richtung personalisierter Präzisionsmedizin erfolgen.

In diesem Beitrag geht es einerseits um die historische Entwicklung von KI in der Medizin sowie um die Frage, wieso bisher in Deutschland eine umfassende Nutzung nicht stattgefunden hat bzw. wo die Herausforderungen liegen, deren Bewältigung KI zum „Gamechanger“ im Gesundheitswesen werden lassen könnte.

## Historische Entwicklung und Meilensteine der KI im Gesundheitswesen

**Abb. 1:**
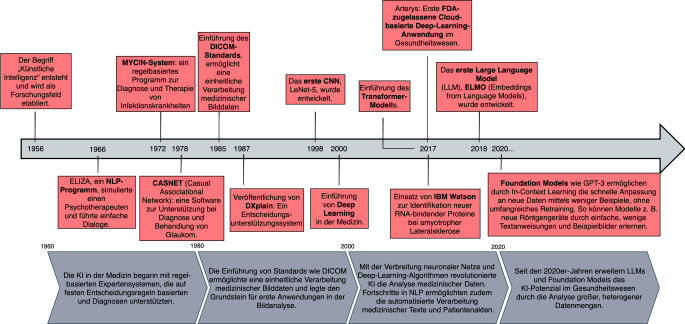
Historische Entwicklung der künstlichen Intelligenz im Gesundheitswesen. Die Abbildung zeigt die Entwicklung von regelbasierten Systemen in den 1960er-Jahren bis hin zu datengetriebenen und lernenden Systemen der Gegenwart. Meilensteine sind u. a. die Einführung des Expertensystems „MYCIN“ (1972), die Entwicklung des Bildstandards „DICOM“ (1985), der Einsatz von „Convolutional Neural Networks“ (CNNs) in der Bildverarbeitung (1998), der Durchbruch von Deep-Learning-Modellen, die Veröffentlichung der Transformer-Architektur (2017), die erste FDA-zugelassene Deep-Learning-Anwendung „Arterys“ (2017) sowie die Etablierung leistungsstarker Foundation Models

Die Entwicklung der KI im Gesundheitswesen reicht von den Anfängen regelbasierter Expertensysteme bis zu modernen Ansätzen des maschinellen Lernens (ML) und tiefen Lernens (Deep Learning, DL). Die Meilensteine und die zeitliche Entwicklung dieser Transformation sind in Abb. 1 dargestellt.

Die ersten Anwendungen der KI im Gesundheitswesen entstanden in den 1960er-Jahren. Ein frühes Beispiel aus dieser Zeit ist der erste entwickelte Chatbot „Eliza“, der einfache psychotherapeutische Gespräche simulierte und die Grundlagen der natürlichen Sprachverarbeitung demonstrierte [1]. Insgesamt sind die Anfänge der KI-Entwicklung von sogenannten Expertensystemen geprägt. Diese computergestützten Systeme basieren auf festen, regelbasierten Entscheidungsprozessen und wurden speziell entwickelt, um medizinische Fachkräfte bei der Diagnosestellung und Therapieplanung zu unterstützen. Eines der ersten Expertensysteme, entstanden in den frühen 1970er-Jahren, ist „MYCIN“, ein System, das Infektionskrankheiten diagnostizierte und therapeutische Vorschläge für die Antibiotikabehandlung lieferte [2]. Ein weiteres erstes Expertensystem aus den 1970er-Jahren ist „CASNET“, ein kausal-assoziatives Netzwerk, das für die Diagnose und Behandlung von Glaukom entwickelt wurde und Ärzten strukturierte Entscheidungshilfen bot [3].

In den 1980er-Jahren führte die steigende Verfügbarkeit medizinischer Daten zur Entwicklung von Standards wie DICOM (Digital Imaging and Communications in Medicine), der die standardisierte Verarbeitung medizinischer Bilddaten ermöglichte und Anwendungen in der Bildverarbeitung begründete. Zeitgleich wurde mit der Einführung von datengetriebenen Ansätzen „DXplain“, ein Entscheidungsunterstützungssystem, das den Übergang von regelbasierten zu datengetriebenen Ansätzen in der klinischen Entscheidungsfindung markiert, veröffentlicht, das auf Grundlage eingegebener Symptome eine differenzialdiagnostische Einschätzung lieferte und als elektronisches medizinisches Nachschlagewerk diente [4]. Ende der 1980er-Jahren erweiterten „Convolutional Neural Networks“ (CNNs) die KI-Forschung erheblich. Als bahnbrechender Fortschritt in der automatisierten Bildanalyse ermöglichten CNNs erstmals eine effektive Verarbeitung komplexer visueller Datenstrukturen und fanden später insbesondere Anwendung bei der Tumorerkennung sowie der computergestützten diagnostischen Entscheidungsunterstützung [5, 6]. „AlexNet“, im Jahr 2012 veröffentlicht, markierte einen Durchbruch in der Bildklassifikation, indem es als erstes tiefes CNN herausragende Ergebnisse erzielte [7].

Seit den 2000er-Jahren markierten Fortschritte im Deep Learning und dem „Natural Language Processing“ (NLP) einen Paradigmenwechsel in der medizinischen KI-Forschung. NLP-Modelle ermöglichten die automatisierte Analyse medizinischer Texte, was Potenzial für die Anwendung in der Auswertung von Arztbriefen und der Kodierung von Diagnosen bot [8]. Auch im Bereich der Bilddatenanalyse entwickelte sich KI weiter. „Arterys“ erhielt 2017 als erste klinische Cloud-basierte Deep-Learning-Anwendung die Zulassung der US-amerikanischen Food and Drug Administration (FDA) – zunächst für die Analyse von kardialen Magnetresonanztomografien (MRT). Später wurde die Anwendung auf die Auswertung von Computertomografien (CT) der Leber, der Lunge und des Kopfes sowie auf Röntgenaufnahmen und andere bildgebende Verfahren ausgeweitet [9]. Im gleichen Jahr wurde „IBM Watson“, ein KI-System mit natürlicher Sprachverarbeitung und Datenanalysefähigkeiten, genutzt, um RNA-bindende Proteine zu identifizieren, die bei amyotropher Lateralsklerose (ALS) eine Rolle spielen [10]. Mit der Veröffentlichung des Papers „Attention is All You Need“ im Jahr 2017 leitete das Transformer-Modell einen fundamentalen Umbruch im Bereich des NLP ein [11]. Es ersetzte herkömmliche sequenzielle Architekturen durch ein auf Attention-Mechanismen basierendes Modell, das erstmals eine flexible und kontextabhängige Verarbeitung von Texten in großem Maßstab ermöglichte. Aufbauend auf diesem Prinzip entstanden leistungsstarke „Large Language Models“ (LLMs) wie „BERT“ und „GPT“, die nicht nur das NLP nachhaltig veränderten, sondern auch weitreichende neue Anwendungsmöglichkeiten in der medizinischen KI eröffneten.

Seit den 2020er-Jahren hat die Weiterentwicklung von Foundation Models, wie „GPT-3“, das Potenzial der KI im Gesundheitswesen deutlich erweitert. Foundation Models bezeichnen großskalige, vortrainierte KI-Modelle, die als universelle Grundlage für eine Vielzahl spezifischer Anwendungen dienen; sie umfassen unter anderem LLMs und unterscheiden sich von klassischen, domänenspezifischen Modellen durch ihre Vielseitigkeit und Adaptionsfähigkeit. Diese Modelle haben das Potenzial, große, heterogene Datensätze zu analysieren und Ärzte bei Diagnose und Therapieplanung zu unterstützen, indem sie medizinische Fachliteratur, Patientenakten und medizinische Leitlinien miteinander verbinden können. Zudem ermöglichen sie die Integration verschiedener Datenquellen, darunter Bild‑, Text- und Genomdaten, in einheitliche klinische Entscheidungssysteme [12]. Durch diese Fortschritte können komplexe Zusammenhänge im medizinischen Kontext greifbarer gemacht werden und bieten die Grundlage für neue Möglichkeiten in der personalisierten und präzisen Gesundheitsversorgung. Der Einsatz von Foundation Models im klinischen Kontext steht noch am Anfang und stellt bislang ein Anwendungspotenzial dar, das durch regulatorische Vorgaben begrenzt ist.

KI im Gesundheitswesen ist durch bedeutende technologische Fortschritte geprägt. Die Integration moderner KI-Technologien in klinische Arbeitsabläufe bietet großes Potenzial zur Verbesserung von Diagnose und Therapie. Die Überführung von Forschungsergebnissen in die klinische Praxis stellt eine zentrale Herausforderung dar.

## Arten von KI und Technologien im Gesundheitswesen

Im Gesundheitswesen finden verschiedene Arten von KI und Technologien Anwendung, die transformative Auswirkungen auf die Bereiche Prävention, Diagnose, Therapie und Administration haben. Eine wesentliche Rolle spielt das maschinelle Lernen, welches in 3 Hauptkategorien unterteilt werden kann: „Unsupervised Learning“ (unüberwachtes Lernen) findet Anwendung in der Mustererkennung, etwa zur Ableitung unbekannter Zusammenhänge aus großen, unstrukturierten Datenmengen. Beim „Supervised Learning“ (überwachtes Lernen) werden gelabelte Daten genutzt, um Klassifikationsprobleme wie die Differenzierung von Krankheitsbildern zu lösen. „Reinforcement Learning“ (verstärkendes Lernen) basiert auf Belohnungsmechanismen und findet insbesondere in der Robotik Anwendung, wo es autonome Entscheidungsprozesse ermöglicht [13].

**Abb. 2:**
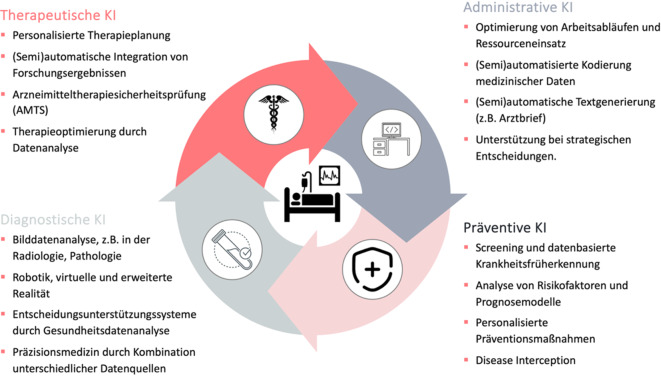
Hauptanwendungsbereiche von künstlicher Intelligenz (KI) im Gesundheitswesen. Die präventive KI zielt auf die Früherkennung und Verhinderung von Krankheiten ab, während die administrative KI organisatorische und logistische Prozesse optimiert. Diagnostische KI unterstützt die präzise Erkennung und Vorhersage von Krankheiten und therapeutische KI ermöglicht die Optimierung personalisierter Behandlungsstrategien

Des Weiteren lässt sich eine Kategorisierung von KI-Technologien im Gesundheitswesen nach ihrem Fokus vornehmen, wobei eine Differenzierung zwischen *schwacher* und *starker* KI erfolgt. Gegenwärtig liegt der Schwerpunkt auf spezialisierter, schwacher KI, während starke KI, die allgemeine Intelligenz umfasst, eine Vision für die Zukunft darstellt. Innerhalb der schwachen KI im medizinischen Anwendungsbereich lassen sich 4 Haupttypen unterscheiden: präventive, diagnostische, KI-gestützte Therapie und administrative KI (Abb. 2).

Die *präventive KI* zielt darauf ab, Krankheiten frühzeitig zu erkennen und ihre Entstehung zu verhindern. Durch den Einsatz fortschrittlicher Algorithmen sind eine präzise Analyse von Risikofaktoren, die Entwicklung personalisierter Präventionsmaßnahmen sowie die frühzeitige Erkennung genetischer oder individueller Risikokonstellationen möglich, um präventiv im symptomfreien Frühstadium eingreifen zu können [14]. Die Verwendung von KI-unterstützten EKG-Systemen (AI-ECG) ermöglicht die frühzeitige Erkennung von Herzpathologien sowie die Prognose von Überlebensraten [15]. Eine Übersichtsarbeit aus dem Jahr 2021 zeigt anhand von über 3,8 Mio. Patientendaten das Potenzial präventiver KI für die frühe Krebsdiagnose durch Risikostratifizierung und Symptomanalyse in der Primärversorgung – ein wichtiger Schritt hin zur Etablierung KI-gestützter Vorsorgestrategien [16].

Die *diagnostische KI* erweitert die medizinische Diagnostik, indem sie Krankheiten präzise und effizient erkennt. Der Einsatz von KI-Technologien erlaubt eine Beschleunigung diagnostischer Prozesse und eine signifikante Steigerung der Genauigkeit von Diagnosen, insbesondere wenn verschiedene Datenquellen kombiniert und somit präzise Entscheidungsunterstützungssysteme entwickelt werden können. Darüber hinaus kann KI auch in Bereichen wie multimodaler Gerätesteuerung, Robotik sowie virtueller und erweiterter Realität zur Unterstützung diagnostischer Verfahren eingesetzt werden, etwa durch die Navigation bei bildgestützten Eingriffen oder die Visualisierung komplexer Daten für die Entscheidungsfindung. Ein Beispiel diagnostischer KI ist die multimodale Überlebensprognose bei Bauchspeicheldrüsenkrebs, die durch die Kombination klinischer, bildgebender und genetischer Daten präzisere Vorhersagen als traditionelle Methoden ermöglicht [17]. Eine Analyse von 83 Studien zeigt, dass generative KI-Modelle eine geringere diagnostische Genauigkeit als Experten, jedoch vergleichbare Ergebnisse mit Nichtexperten erzielen. Dies unterstreicht ihr Potenzial als unterstützendes Werkzeug in klinischen Entscheidungssituationen, insbesondere in ressourcenschwachen oder nichtspezialisierten Kontexten [18].

Der Einsatz *KI-gestützter Therapie* eröffnet neue Möglichkeiten zur individualisierten und effektiveren Behandlung von Patienten. Durch den Einsatz innovativer Algorithmen kann eine bessere Abstimmung von Therapien auf die individuellen Bedürfnisse von Patienten gewährleistet werden, was zu optimierten Ergebnissen für die Patienten führt. Gleichzeitig tragen diese Technologien zur effizienteren Nutzung medizinischer Ressourcen und zur Verbesserung der Versorgungsqualität bei, beispielsweise ein Deep-Learning-Modell, welches personalisierte Risikoprognosen für Thrombozytentransfusionen erstellt, wodurch die Ressourcenplanung optimiert und Versorgungsengpässe verhindert werden können [19]. Eine Metaanalyse randomisierter Studien zeigt, dass multimodale, mobil integrierte und generative KI-Chatbots Symptome von Depression und Stress signifikant reduzieren und dabei mittlere bis große Effekte erzielen [20].

Die Implementierung von KI im *administrativen* Sektor des Gesundheitswesens führt zu einer Transformation der organisationalen und logistischen Strukturen medizinischer Einrichtungen. Der Einsatz intelligenter Systeme erlaubt eine optimierte Gestaltung von Arbeitsabläufen, eine effiziente Nutzung von Ressourcen sowie eine solidere Grundlage für strategische Entscheidungen. Dies fördert eine Steigerung der Qualität und Wirtschaftlichkeit der Gesundheitseinrichtungen. Die KI-gestützte Krankenhausverwaltung kann dem Management Prognosen und Analysen zur Verfügung stellen, beispielsweise zur Bettenbelegung oder Personalplanung. Dadurch werden strategische Entscheidungen optimiert und Ressourcen effektiver eingesetzt [21]. Eine systematische Übersichtsarbeit analysiert 70 Studien, weist jedoch darauf hin, dass quantitative Aussagen zur Effektivität von KI-Anwendungen im administrativen Gesundheitswesen bislang selten sind [22].

## Herausforderungen und Limitationen

Durch den Einsatz komplexer KI werden auch (neue) ethische und soziotechnische Fragen aufgeworfen. Kritische Punkte sind zum Beispiel fehlende Transparenz und Nachvollziehbarkeit, sodass es zu „Blackbox“-Problemen kommen kann. Hierbei lässt sich für Außenstehende nicht mehr nachvollziehen, wie das KI-Modell zu seinem Ergebnis gekommen ist. Diese Blackbox-Problematik kann auch die Zuschreibung moralischer Verantwortung beeinflussen [23–25]. Dem medizinischen Personal die volle moralische Verantwortung für nicht erkannte Fehler einer KI zuzuweisen, scheint problematisch. Denn um Fehler der KI zu erkennen, ist ein immer komplexer werdendes technisches Verständnis notwendig [24].

Zurzeit ist das ärztliche Personal dazu angehalten, stets eine Plausibilitätsprüfung der durch die KI ermittelten Ergebnisse durchzuführen [25]. Inwiefern sich diese Forderung langfristig aufrechterhalten lässt, ist fraglich. Zum einen besteht das Risiko eines „Automation Bias“. Hierbei werden die Vorhersagen des KI-Modells ohne weitere Überprüfung als richtig angesehen und als Basis für weitere Handlungen genommen. Mögliche Fehler der KI werden nicht mehr in Betracht gezogen. Eine Möglichkeit, dem Automation Bias zu begegnen, kann „Explainable AI“ sein. Wenn diese laienverständlich die jeweiligen Quellen oder Berechnungsschritte ihrer Ergebnisse darstellen, ist es einfacher, diese auf Fehler zu prüfen. Zum anderen besteht langfristig die Gefahr eines Fähigkeitsverlusts bei Gesundheitsfachkräften, wenn KI-Modelle zunehmend medizinisch-technische Tätigkeiten übernehmen. Der damit einhergehende Verlust ärztlichen Erfahrungswissens könnte nicht nur die Überprüfung von KI-Ergebnissen erschweren, sondern auch das Arzt-Patienten-Verhältnis beeinträchtigen [25].

Darüber hinaus bringt der Einsatz von KI in der Medizin zahlreiche weitere ethische und soziotechnische Herausforderungen mit sich, deren Diskussion in diesem Rahmen nicht geleistet werden kann. Hierzu zählen beispielsweise die Akzeptanz von KI im Behandlungsprozess seitens des Fachpersonals [26] *und* der Patienten, eine mögliche Dehumanisierung der medizinischen Profession durch die bloße Kategorisierung von Daten [27] oder Fragen der gerechten Verteilung von Ressourcen zur Finanzierung von KI-Entwicklung und herkömmlicher medizinischer Forschung [27].

Neben den ethischen und soziotechnischen Herausforderungen, die der Einsatz komplexer KI-Modelle in der Medizin mit sich bringt, ergeben sich auch regulatorische Herausforderungen. In seiner aktuellen Form steht der „Artificial-Intelligence-Act“ (AI-Act) der Europäischen Union (EU) in einem Spannungsverhältnis zur Datenschutzgrundverordnung (DSGVO) und der Medical-Device-Regulation (MDR) im medizinischen Bereich. So verbietet die DSGVO beispielsweise die Verarbeitung sensibler Daten wie Religionszugehörigkeit, Alter und ethnische Herkunft, während der AI-Act die Verarbeitung der Daten in Ausnahmefällen gestattet [28].

Die Zulassung unter der MDR ist die Voraussetzung für die Anwendung eines KI-Modells in der Medizin. Sobald eine KI-Anwendung sowohl durch den AI-Act als auch durch die MDR als Hochrisikoanwendung eingestuft ist, müssen die Anforderungen beider Verordnungen erfüllt werden. Da nicht klar ist, ob eine der beiden Verordnungen vorrangig vor der anderen zu beachten ist, kommt es zu Unsicherheiten und doppelter Zertifizierungs- und Dokumentationsarbeit. Außerdem stellen die begrenzten Kapazitäten der zuständigen Zulassungsstellen für neue KI-Modelle die Unternehmen vor Herausforderungen [28].

KI in der Medizin ist weiterhin mit einigen technischen Herausforderungen verbunden. Vor allem die Unvermeidbarkeit von „Halluzinationen“ in generativen Modellen (Ausgabe von Informationen, die faktisch falsch, erfunden oder unbelegt sind) erfordert Risikoabschätzungen, die den praktischen Einsatz insbesondere in hochsensiblen diagnostischen oder therapeutischen Bereichen einschränken können. Weitere kritische Aspekte sind der Bias in Trainingsdaten, der die Übertragbarkeit und Generalisierbarkeit der Modelle beeinträchtigt, sowie die erheblichen Herausforderungen hinsichtlich der Implementierungs- und Betriebskosten. Im Gegensatz dazu bieten wissens- und logikbasierte KI-Ansätze inhärente Vorteile in Bezug auf Erklärbarkeit, Nachvollziehbarkeit und technische Anforderungen. Daher ist eine Abwägung zwischen Deep-Learning-Ansätzen und klassischen regelbasierten Ansätzen in individuellen medizinischen Kontexten sinnvoll.

Eine weitere wesentliche Herausforderung für KI in der Medizin ist die anhaltende Fragmentierung von Gesundheitsdaten durch die bestehenden Datensilos. Hinzu kommt eine fehlende Standardisierung, obwohl mit der Gesundheits-IT-Interoperabilitäts-Governance-Verordnung [29] klare Vorgaben zur Förderung von semantischer und syntaktischer Interoperabilität in Deutschland bestehen. Diese ließe sich beispielsweise auf bereits zur Verfügung stehenden international anerkannten Standards aufbauen. Die fehlende Interoperabilität der Daten und die bestehenden Datensilos wirken sich nachteilig auf die Verfügbarkeit von Trainingsdaten für die KI-Entwicklung aus. Auch die konkrete Anwendbarkeit von KI-Anwendungen kann in Mitleidenschaft gezogen werden, wenn bspw. unterschiedliche Gesundheitsdaten aus unterschiedlichen Silos kombiniert werden müssen. Weitere Probleme ergeben sich für KI-Anwendungen, wenn Daten nur in unstrukturierter Form vorliegen [30]. Wünschenswert wäre daher die Harmonisierung der heterogenen Gesundheitsdatensets durch einheitliche Datenstandards wie „Fast Healthcare Interoperability Resources“ (FHIR). Darüber hinaus sollten internationale Nomenklaturen wie „SNOMED CT“ und „LOINC“ umgesetzt werden, um die semantische und syntaktische Interoperabilität der Daten sicherzustellen [31].

## Erfolgsmodell: Das „Smart Hospital“

**Abb. 3:**
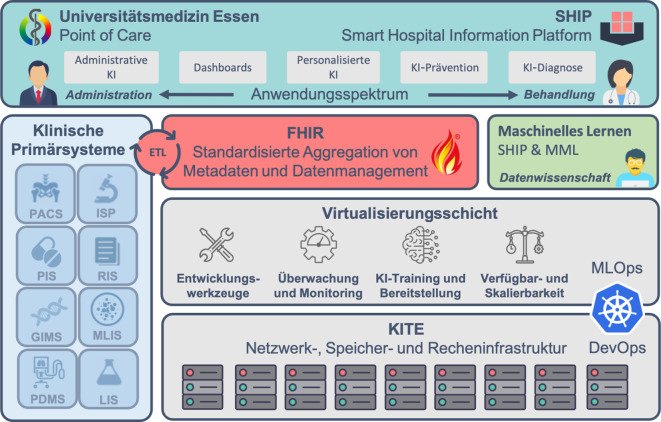
Schichtenmodell des „Smart Hospital Essen“. Klinische Primärsysteme (z. B. PACS, RIS, PDMS) werden über FHIR standardisiert und nach dem ETL-Prinzip (engl. für Extract, Transform, Load) aggregiert. Dies bildet die Grundlage für Anwendungsentwicklung und KI-Forschung. Durch das KITE-Projekt entsteht eine skalierbare Infrastruktur für die Bereitstellung von KI-Anwendungen am Point of Care. Die Teams von SHIP und MML setzen Forschungsergebnisse direkt in klinische Anwendungen um. Die Anwendungen decken das gesamte Spektrum von der administrativen KI bis hin zur KI-gestützten Diagnose ab

Die Universitätsmedizin Essen (UME) verfolgt seit 2015 das Konzept eines „Smart Hospital“ und gilt als Beispiel für eine erfolgreiche Umsetzung der Digitalisierung im Gesundheitswesen. Ziel ist die Verbesserung der Patientenversorgung durch intelligente, digitale Vernetzung klinischer Prozesse. Im Rahmen dieser Strategie wurden die Projekte „Smart Hospital Information Platform“ (SHIP; [32]) und „KI-Translation Essen“ (KITE) initiiert. SHIP dient als Referenzarchitektur für die Integration von Gesundheitsdaten, während KITE darauf abzielt, KI in den klinischen Alltag zu bringen. Wie in Abb. 3 dargestellt, sind beide Projekte stark miteinander verzahnt und abgestimmt.

Als standardisiertes Architekturmodell bietet SHIP einen Referenzrahmen für die Entwicklung und Implementierung interoperabler Systeme im Gesundheitswesen. Durch branchenspezifische Best Practices schafft SHIP ein gemeinsames Verständnis von Prozessen, Datenstrukturen und zugrunde liegenden Technologien. Klinische Primärsysteme wie PACS, RIS oder PDMS werden – entsprechend den Interoperabilitätsstandards der Medizininformatik-Initiative (Kerndatensatz) – mittels FHIR, einem internationalen Standard für den Austausch medizinischer Daten, standardisiert aggregiert. Anfang 2025 verzeichnet die hauseigene FHIR-Plattform über 1,6 Bill. Ressourcen und verwaltet mehr als 1,5 Mio. Patienten, bei täglich neu hinzukommenden 600.000 Ressourcen. Die lokale IT-Infrastruktur (On-Premise-Infrastruktur) von KITE unterstützt die Anwendung moderner Entwicklungspraktiken wie „DevOps“ (Development and Operations – Integration von Softwareentwicklung und IT-Betrieb) und „MLOps“ (Machine Learning Operations – Integration von maschinellem Lernen in Entwicklungs- und Betriebsprozesse). Diese Ansätze sind entscheidend für die kontinuierliche Entwicklung, Bereitstellung und Wartung von Software und KI-Modellen.

## Trends und zukünftige Entwicklungen

In den letzten 10 Jahren wurden auf dem Gebiet des maschinellen Lernens erhebliche Fortschritte erzielt, vor allem durch die Entwicklung von spezialisierten Modellen. So können beispielsweise Bildmodelle radiologische Aufgaben wie die Erkennung von Lungenentzündungen [33] oder Tumoren [34] mit einer hohen Präzision lösen. Dennoch stoßen diese spezialisierten Ansätze in der Praxis an Grenzen, die ihre Einsatzmöglichkeiten einschränken.

Ein zentrales Problem besteht darin, dass KI-Modelle häufig isoliert arbeiten und keine Informationen aus anderen Modalitäten, wie Textdaten aus elektronischen Patientenakten, genetische Daten oder Laborergebnisse, in ihre Analysen einbeziehen. Zudem fehlt spezialisierten Modellen die Fähigkeit, dynamisch Entscheidungen zu treffen, etwa welches spezialisierte Modell für eine spezifische Aufgabe herangezogen werden sollte. Um Ärzte effektiv zu unterstützen, müsste eine KI flexibel auf Rückfragen eingehen können und eigenständig komplexere Aufgabenstellungen in lösbare Unteraufgaben einteilen.

Mögliche Lösungen zeichnen sich durch den Einsatz von Foundation-Modellen und Agenten ab [35]. Foundation-Modelle ermöglichen multimodales Lernen, indem sie Daten aus verschiedenen Quellen – etwa Text, Bilder und Genomik – integrieren und ganzheitlich analysieren können. LLM-basierte Agenten könnten zudem komplexe Aufgaben orchestrieren, indem sie nicht nur angemessene Modelle für spezifische Aufgaben auswählen, sondern auch eine intuitive Mensch-Maschine-Interaktion ermöglichen [36]. Diese Kombination könnte die Effizienz klinischer Prozesse erheblich steigern, die Arbeitsbelastung von Ärzten reduzieren und die Qualität der Patientenversorgung verbessern [36].

Ein KI-System aus Foundation-Modellen und Agenten könnte beispielsweise in der Notfallmedizin helfen, indem es alle verfügbaren Informationen wie Anamnese, Laborwerte und bildgebende Verfahren integriert. Der Agent steuert die Modelle, z. B. zur Analyse von Röntgenaufnahmen auf Lungenerkrankungen und der Patientenakte zur Identifikation von Risikofaktoren. Er konsolidiert die Ergebnisse und schlägt eine vorläufige Diagnose oder weitere Untersuchungen vor, wobei er dem medizinischen Personal für Rückfragen zur Verfügung steht.

Die Evaluation spezialisierter KI ist durch klar definierte Aufgaben und Datensätze vergleichsweise einfach, während die Bewertung vielseitiger Agenten komplex bleibt und häufig manuelle Expertenprüfung erfordert. In der Zukunft wird entscheidend sein, neue und effizientere Methoden zur Evaluation solcher vielseitigen Systeme zu entwickeln.

## Diskussion

Die historische Entwicklung der KI zeigt eine Transformation der Forschung zur Anwendung der KI im medizinischen Kontext. Aktuelle Trends verdeutlichen die Bedeutung von KI in Bereichen wie Diagnostik, Therapie, Prävention und Administration. Dennoch stehen der Integration von KI-Technologien erhebliche Herausforderungen gegenüber. Zukünftige Forschung sollte sich auf die Schaffung interoperabler Standards, die Implementierung KI-gestützter Systeme in die klinische Routine und die Überwindung bestehender Barrieren wie Datensilos und regulatorischer Herausforderungen konzentrieren. Ein zentraler Fokus liegt auf der Verbesserung der Datenqualität und der Interoperabilität, um eine nahtlose Integration verschiedener medizinischer Datentypen wie Bildgebung, Genomdaten und klinische Freitexte zu ermöglichen. Fortschritte in diesen Bereichen sind essenziell, um die Leistungsfähigkeit KI-gestützter Systeme zu steigern und eine umfassende Unterstützung in der Diagnostik, Therapie und Prävention zu gewährleisten.

Nationale Datenregister und standardisierte Rahmenbedingungen könnten den Austausch zwischen verschiedenen Sektoren fördern und die Grundlage für KI-gestützte Anwendungen schaffen. Initiativen wie der „European Health Data Space“ dienen als beispielhafte Ansätze, um eine vernetzte und sichere Nutzung von Gesundheitsdaten auf europäischer Ebene zu ermöglichen. Diese Reformen und Infrastrukturverbesserungen sind essenziell, um Datenbarrieren zu überwinden und die Potenziale von KI in der medizinischen Versorgung voll auszuschöpfen. Ein Schritt hin zu einer solchen Infrastruktur ist die Ende April 2025 eingeführte, standardisierte und interoperable elektronische Patientenakte (ePA). Diese hat das Potenzial, die derzeitige Fragmentierung der Gesundheitsdatenlandschaft zu überwinden. Eine strukturierte ePA als zentraler Datenspeicher würde die Grundlage für KI-Anwendungen zur effizienteren und personalisierten Versorgung schaffen.

Die Weiterentwicklung der KI im Gesundheitswesen zielt auf eine sichere und effektive Integration ab. Ein zentraler Schwerpunkt liegt auf der Verbesserung der Erklärbarkeit und Transparenz von KI-Modellen, um ihre Entscheidungsprozesse für Ärzte und Patienten nachvollziehbar zu gestalten. Dies ist essenziell, um Vertrauen in KI-gestützte Systeme zu fördern und deren klinische Akzeptanz zu steigern. Ein weiterer Fokus liegt auf der Weiterentwicklung und Implementierung ethischer Leitlinien, die Aspekte wie den Schutz von Patientendaten, algorithmische Fairness und die Vermeidung von Diskriminierung adressieren. Der Fokus auf diese Themen gewährleistet eine verantwortungsvolle Anwendung von KI-Technologien.

Abschließend lässt sich festhalten, dass zukünftige Forschungs- und Entwicklungsansätze verstärkt auf die Förderung von Kooperationen zwischen Anwendern, Forschung, Politik und Industrie sowie auf die Standardisierung von Prozessen und Daten abzielen müssen. Nur so kann das volle Potenzial von KI im Gesundheitswesen sicher und nachhaltig ausgeschöpft werden.

## Fazit

Künstliche Intelligenz birgt erhebliches Potenzial zur Verbesserung der Versorgungsqualität und -effizienz im Gesundheitswesen. Um dieses Potenzial auszuschöpfen, müssen jedoch Herausforderungen wie Datenfragmentierung, mangelnde Interoperabilität, algorithmische Verzerrungen und ethische Fragestellungen adressiert werden. Zentrale Voraussetzungen sind die Entwicklung standardisierter, sicherer Dateninfrastrukturen sowie eine enge Zusammenarbeit zwischen klinischer Praxis, Forschung, Industrie und Politik. Zukünftige Entwicklungen wie Foundation Models und multimodale Datenintegration könnten die Basis für eine stärker personalisierte und prädiktive Medizin schaffen.

## Abkürzungen

CNN: Convolutional Neural Network
DL: Deep Learning
DevOps: Development and Operations
DICOM: Digital Imaging and Communications in Medicine
DSGVO: Datenschutzgrundverordnung
ETL: Extract, Transform, Load
ePA: elektronische Patientenakte
FHIR: Fast Healthcare Interoperability Resources
GIMS: Genetic Information Management System
ISP: Information System Pathology
KI: Künstliche Intelligenz
KITE: KI-Translation Essen
LIS: Laboratory Information System
LLM: Large Language Model
MDR: Medical-Device-Regulation
ML: Machine Learning
MLIS: Microbiological Laboratory Information System
MLOps: Machine Learning Operations
MML: Medical Machine Learning
NLP: Natural Language Processing
PACS: Picture Archiving and Communication System
PDMS: Patient Data Management System
PIS: Pharmacy Information System
RIS: Radiology Information System
SHIP: Smart Hospital Information Platform

## References

[1] Weizenbaum J (1966) ELIZA—a computer program for the study of natural language communication between man and machine. Commun ACM 9(1):36–45

[2] Shortliffe EH (1976) Computer-Based Medical Consultation: MYCIN. Elsevier, New York

[3] Weiss S, Kulikowski CA, Safir A (1978) Glaucoma consultation by computer. Comput Biol Med 8(1):25–40 620517 10.1016/0010-4825(78)90011-2

[4] Barnett GO, Cimino JJ, Hoffer EP (1987) DXplain. An evolving diagnostic decision-support system. JAMA 258(1):67–74 3295316 10.1001/jama.258.1.67

[5] Hosny A, Parmar C, Quackenbush J, Schwartz LH, Aerts HJWL (2018) Artificial intelligence in radiology. Nat Rev Cancer 18(8):500–510. 10.1038/s41568-018-0016-5 29777175 10.1038/s41568-018-0016-5 PMC6268174

[6] LeCun Y, Bottou L, Bengio Y, Haffner P (1998) Gradient based learning applied to document recognition. Proc IEEE 86(11):2278–2324

[7] Krizhevsky A, Sutskever I, Hinton GE (2012) Imagenet classification with deep convolutional neural networks. Adv Neural Inf Process Syst 25:

[8] Velupillai S, Mowery D, South BR, Kvist M, Dalianis H (2015) Recent advances in clinical natural language processing in support of semantic analysis. Yearb Med Inform 24(1):183–193. 10.15265/IY-2015-009 10.15265/IY-2015-009 PMC4587060 26293867

[9] Arterys: medical imaging cloud AI. https://arterys.com/. Zugegriffen: 10. Dez. 2024

[10] Bakkar N, Kovalik T, Lorenzini I et al (2018) Artificial intelligence in neurodegenerative disease research: use of IBM Watson to identify additional RNA-binding proteins altered in amyotrophic lateral sclerosis. Acta Neuropathol 135(2):227–247. 10.1007/s00401-017-1785-8 29134320 10.1007/s00401-017-1785-8 PMC5773659

[11] Vaswani A, Shazeer N, Parmar N et al (2017) Attention is All you Need. Adv Neural Inf Process Syst 30:

[12] Moor M, Banerjee O, Abad ZSH et al (2023) Foundation models for generalist medical artificial intelligence. Nature 616(7956):259–265. 10.1038/s41586-023-05881-4 37045921 10.1038/s41586-023-05881-4

[13] Jo T (2021) Machine Learning Foundations: Supervised, Unsupervised, and Advanced Learning. Springer, Cham

[14] Wiese L, Diehl A, Huster S (2024) Disease Interception als Chance und Herausforderung: Eine interdisziplinäre Analyse. Nomos, Baden-Baden

[15] Yu SY, Li HL, Tse AK et al (2023) Pre-admission and In-Hospital Statin Use is Associated With Reduced Short-Term Mortality in Infective Endocarditis. Mayo Clin Proc 98(2):252–265 36114025 10.1016/j.mayocp.2022.06.006

[16] Jonas OT, Calanzani N, Saji S et al (2021) Artificial Intelligence Techniques That May Be Applied to Primary Care Data to Facilitate Earlier Diagnosis of Cancer: Systematic Review. J Med Internet Res 23(3):e23483 33656443 10.2196/23483 PMC7970165

[17] Keyl J, Kasper S, Wiesweg M et al (2022) Multimodal survival prediction in advanced pancreatic cancer using machine learning. ESMO Open 7(5):100555. 10.1016/j.esmoop.2022.100555 35988455 10.1016/j.esmoop.2022.100555 PMC9588888

[18] Takita H et al (2025) A systematic review and meta-analysis of diagnostic performance comparison between generative AI and physicians. NPJ Digit Med 8(1):175. 10.1038/s41746-025-01543-z 40121370 10.1038/s41746-025-01543-z PMC11929846

[19] Engelke M, Schmidt C, Baldini G (2023) Optimizing platelet transfusion through a personalized deep learning risk assessment system for demand management. Blood 142(26):2315–2326. 10.1182/blood.2023021172 37890142 10.1182/blood.2023021172

[20] Li H, Zhang R, Lee YC, Kraut RE, Mohr C (2023) Systematic review and meta-analysis of AI-based conversational agents for promoting mental health and well-being. NPJ Digit Med 6(1):236. 10.1038/s41746-023-00979-5 38114588 10.1038/s41746-023-00979-5 PMC10730549

[21] Alves M, Seringa J, Silvestre T, Magalhães T (2024) Use of Artificial Intelligence tools in supporting decision-making in hospital management. BMC Health Serv Rev 24(1):1282. 10.1186/s12913-024-11602-y 10.1186/s12913-024-11602-y PMC11515352 39456040

[22] Caruana A, Bandara M, Musial K, Catchpoole D, Kennedy PJ (2018) Machine learning for administrative health records: A systematic review of techniques and applications. Artif Intell Med 144:102642. 10.1016/j.artmed.2023.102642 10.1016/j.artmed.2023.102642 37783537

[23] Cockelbergh M (2020) AI Ethics. MIT Press

[24] Smith H, Birchley G, Ives J (2024) Artificial intelligence in clincal decision-making: Rethinking personal moral responsibility. Bioethics 38(1):78–86. 10.1111/bioe.13222 37724044 10.1111/bioe.13222 PMC10953430

[25] Zentrale Ethikkommission der Bundesärztekammer (2021) Entscheidungsunterstützung ärztlicher Tätigkeit durch Künstliche Intelligenz. Dtsch Ärztebl 118(33–34):A1–A13

[26] Lambert SI, Madi M, Sopka S et al (2023) An integrative review on the acceptance of artificial intelligence among healthcare professionals in hospitals. NPJ Digit Med 6(1):111. 10.1038/s41746-023-00852-5 37301946 10.1038/s41746-023-00852-5 PMC10257646

[27] Morley J, Floridi L (2024) The Ethics of AI in Health Care: An Updated Mapping Review. 10.2139/ssrn.4987317

[28] Hacker P (2024) Der AI Act im Spannungsfeld von digitaler und sektoraler Regulierung 10.11586/2024189

[29] Bundesministerium für Gesundheit (2024) Gesundheits-IT-Interoperabilitäts-Governance-Verordnung. IOP-Governance-Verordnung – GIGV

[30] Dos PSD, Baeßler B (2018) Big data, artificial intelligence and structured reporting. Eur Radiol Exp 2(1):42. 10.1097/ICU.0000000000000676 30515717 10.1186/s41747-018-0071-4 PMC6279752

[31] Wang SY, Pershing S, Lee AY (2020) Big data requirements for artificial intelligence. Curr Opin Ophthalmol 31(5):318–323 32657996 10.1097/ICU.0000000000000676 PMC8164167

[32] - (2025) Künstliche Intelligenz in der Medizin. Dtsch Ärztebl Int, S 238

[33] Ko H, Chung H, Kang WS et al (2020) COVID-19 pneumonia diagnosis using a simple 2D deep learning framework with a single chest CT image: Model development and validation. J Med Internet Res 22(6) 10.2196/19569 PMC7332254 32568730

[34] Chatterjee S, Nizamani FA, Nürnberger A, Speck O (2022) Classification of brain tumours in MR images using deep spatiospatial models. Sci Rep 12(1):1505. 10.1038/s41598-022-05572-6 35087174 10.1038/s41598-022-05572-6 PMC8795458

[35] Moor M, Banerjee O, Abad ZSH et al (2023) Foundation models for generalist medical artificial intelligence. Nature 616(7956):259–265. 10.1038/s41586-023-05881-4 37045921 10.1038/s41586-023-05881-4

[36] Qiu J, Lam K, Li G et al (2024) LLM-based agentic systems in medicine and healthcare. Nature. Mach Intell 6(12):1418–1420. 10.1038/s42256-024-00944-1

